# Sequence-based identification of interface residues by an integrative profile combining hydrophobic and evolutionary information

**DOI:** 10.1186/1471-2105-11-402

**Published:** 2010-07-28

**Authors:** Peng Chen, Jinyan Li

**Affiliations:** 1 Bioinformatics Research Center, School of Computer Engineering, Nanyang Technological University, 639798 Singapore

## Abstract

**Background:**

Protein-protein interactions play essential roles in protein function determination and drug design. Numerous methods have been proposed to recognize their interaction sites, however, only a small proportion of protein complexes have been successfully resolved due to the high cost. Therefore, it is important to improve the performance for predicting protein interaction sites based on primary sequence alone.

**Results:**

We propose a new idea to construct an integrative profile for each residue in a protein by combining its hydrophobic and evolutionary information. A support vector machine (SVM) ensemble is then developed, where SVMs train on different pairs of positive (interface sites) and negative (non-interface sites) subsets. The subsets having roughly the same sizes are grouped in the order of accessible surface area change before and after complexation. A self-organizing map (SOM) technique is applied to group similar input vectors to make more accurate the identification of interface residues. An ensemble of ten-SVMs achieves an MCC improvement by around 8% and F1 improvement by around 9% over that of three-SVMs. As expected, SVM ensembles constantly perform better than individual SVMs. In addition, the model by the integrative profiles outperforms that based on the sequence profile or the hydropathy scale alone. As our method uses a small number of features to encode the input vectors, our model is simpler, faster and more accurate than the existing methods.

**Conclusions:**

The integrative profile by combining hydrophobic and evolutionary information contributes most to the protein-protein interaction prediction. Results show that evolutionary context of residue with respect to hydrophobicity makes better the identification of protein interface residues. In addition, the ensemble of SVM classifiers improves the prediction performance.

**Availability:**

Datasets and software are available at http://mail.ustc.edu.cn/~bigeagle/BMCBioinfo2010/index.htm.

## Background

In living cells, proteins interact with other proteins in order to perform specific biological functions, such as signal transduction or immunological recognition, DNA replication and gene translation, as well as protein synthesis [1]. These interactions are localized to the so-called "interaction sites" or "interface residues".

Identification of these residues will allow us to understand how proteins recognize other molecules and to gain clues into their possible functions at the level of the cell and at the organism. It can also improve our understanding on disease mechanisms and further advance pharmaceutical design [2,3]. 3D (three-dimensional) structures of proteins are the basis for the identification. However, resolving 3D protein structures by experimental methods, such as X-ray crystallography and nuclear magnetic resonance, is much more time-consuming than sequencing proteins. This is the reason why less than 62300 protein structures are available in PDB databank [4] while more than ten million proteins are sequenced in the UniProtKB/TrEMBL database [5], as of Jan. 2010. To narrow the huge gap, various computational methods have been developed to predict protein structures, assisted by the abundance of protein information deposited in various biological databases. Among them, methods to identify protein-protein interface residues have attracted research attention for a long time.

The pioneering work by Kini and Evans addressed the issue of protein interaction site prediction by a unique predictive method based on the observation that "proline" is the most common residue found in the flanking segments of interaction sites [6]. Jones and Thornton were aimed to analyze [7] and predict [8] surface patches that overlap with interfaces by computing a combined score that gives the probability of a surface patch forming protein-protein interactions. Other works have addressed various aspects of protein structure and behavior, such as detecting patch analysis [9], solvent-accessible surface area buried upon association [10], free energy changes upon alanine-scanning mutations [11], in silico two hybrid systems [12], sequence or structure conservation information [13-17], and sequence hydrophobicity distribution [18].

Among them, many machine learning methods have been developed or adopted, such as those using support vector machine (SVM) [16,17,19-22], neural network [13-15,23,24], genetic algorithm [25,26], hidden Markov models [27], Bayesian networks [28,29], random forests [30,31], and so on.

Numerous properties were used in previous work to identify protein-protein interactions. They can be roughly divided into two categories: sequence-based properties and structure-based properties. Sequence-based properties include residue composition and propensity [7,22], hydrophobic scale [32], predicted structural features such as predicted secondary structures [24], features from multiple sequence alignments [17,33], and so on [34]. On the other hand structure-based properties were also widely utilized, such as the size of interfaces [7,35], shape of interfaces [36-38], clustering of interface atoms [39,40], B-factor [21], electrostatic potential [19,21], spatial distribution of interface residues [39,40], and others [41]. The existing methods using these properties showed good performance in the prediction of protein-protein interactions. However, those properties that are specifically significant for particular protein complexes have not been fully assessed. Furthermore, a large set of properties do not always perform well.

Since the amount of protein structures is significantly smaller than those of protein sequences determined by large-scale DNA sequencing methods, it is important to identify protein-protein interaction sites from amino acid sequences alone. It is also valuable to use sequence-based features without experimental 3D structure information. Actually, predicted structure features such as secondary structure can still be helpful to the identification of interaction sites [34]. However, sequence based approaches to identify protein interaction sites are still more difficult to those based on structure information. The reasons are in that: (1) the relationship between sequence-based features and protein-protein interactions are not fully understood; (2) how to represent each residue in a protein by a series of sequence-based features is difficult; (3) the unbalanced data between interaction samples and non-interaction samples may worsen the interface identification [30].

This work addresses these issues by integrative features and by adopting an SVM ensemble method based on balanced training datasets. Since identification of interaction sites in hetero-complexes are much more difficult and more interesting than that in homo-complexes, in this work we focus on hetero-complexes. We first design a schema to represent each residue that integrates hydrophobic and evolutionary information of the residue in a complex. Then an ensemble of SVMs is developed, where SVMs train on different pairs of positive (interface samples) and negative (non-interface samples) subsets. The subsets having roughly the same sizes are grouped in the order of accessible surface area change (ΔASA) before and after complexation. A self-organizing map (SOM) technique [42] is applied to group similar training samples. This is aimed to make more accurate the identification of interface residues. An ensemble of ten-SVMs achieves an MCC improvement by around 8% and F1 improvement by around 9%, compared to those by three-SVMs. We also found that the SVMs ensemble always performs better than individual SVMs. Moreover, using SOM technique achieves an increase of MCC by 1.3 and an increase of F1 by 2%.

## Results

We calculated amino acid composition in our dataset to show the propensity information of the 20 amino acid types between interface and non-interface regions. The propensities for the 20 amino acid types in a logarithm (*log*_2_) scale are shown in Additional file 1. Results show that amino acids with smaller propensity values, such as 'A', 'G', and 'V', representing hydrophobicity, are always involved in non-interface regions. Conversely, hydrophilic amino acids 'R', 'Y', 'W', and 'H' often present in interface regions. Some of these discoveries are consistent with other literature [18,43]. Interestingly, Arginine is the most frequently occurring residue in interface regions while Cysteine and Alanine appear in non-interface regions mostly.

### Determination of the sliding window length

A sliding window technique is used to represent each target residue in this study, where the most challenging issue is to represent each residue by a feature vector and further to construct a predictor. Our first step is the determination of a good sliding window length since prediction performance is usually varied with window length *L*. The tradeoff between prediction performance and the algorithm complexity is also concerned. In this work three individual SVMs were selected from the ten-SVMs without SOM and therefore 120 possible combinations were obtained. The average performance of those SVMs was used to determine the window length. Here five levels of window length, 5, 11, 15, 19, and 27 were attempted. Results show that a sliding window with 19 residues is sufficient to train and test our model, although the model with a window length 27 performed a little better than that with a window length 19. However, the model performed faster than that with the window length 27. The comparison of sensitivity-precision under different window lengthes is illustrated in Additional file 2. Note that using a window length 5 leads to the worst performance. If not otherwise stated in this work, we adopt the window length 19 to evaluate our model and identify protein-protein interface residues.

### Prediction performance without SOM

Additional file 3 shows the performance comparison among the combined SVMs as discussed above with three thresholds. Because none of single measures can fully evaluate prediction performance, we just show all the evaluations on our predictor under six measurements. In this work, MCC and F1 are used as the main measures to evaluate our method. Actually using MCC as a benchmark measurement may lead to cover less positive samples, while using F1 to achieve balanced performance between sensitivity and precision measures may lead to truly identify less positive samples. From this figure, SVM with threshold 3 performs better than those with thresholds 1 and 2, and achieves a sensitivity of 31.39%, precision of 81.12%, specificity of 96.74%, accuracy of 76.6%, and F1 of 45.27% when reaching the largest MCC of 0.4009. In the case of benchmark measurement of F1, additionally, our model with threshold 3 achieve s a sensitivity of 78.44%, precision of 46.79%, specificity of 60.26%, accuracy of 65.86%, MCC of 0.3576, when reaching the largest F1 of 58.62%.

**Figure 1:**
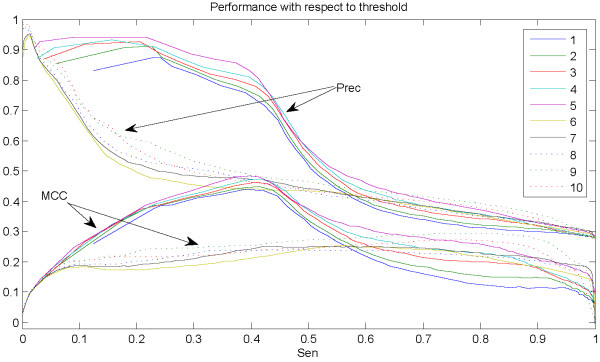
**Performance by our model without SOM**. The figure illustrates the performance curves of sensitivity-precision and sensitivity-MCC after combining the ten-SVMs. Numbers in the legend stand for SVM with different thresholds. Note that curves with the same color correspond to the model with the same threshold.

To fully understand the power of our method, we investigate the combination of all the ten-SVMs. Figure 1 shows the performance comparison of the ten combined SVMs with different thresholds. The two types of performance curves illustrate the performance of sensitivity-precision and that of sensitivity-MCC, respectively. Results from Figure 1 show that the 5-th combined SVM outperforms models with other thresholds. Furthermore, combined SVMs with thresholds from 1 to 5 perform better than those with thresholds from 6 to 10 (particularly when achieving sensitivity of less than 55%), while individual combined SVM within the former or the latter groups yields similar prediction performance. However, combined SVMs yield similar precisions but different MCCs when achieving sensitivities of more than 55%. Table 1 shows performance comparison of the ten-SVMs before and after the combination. As for the ten-SVMs before combination, the first five SVMs perform better than the latter five ones probably because the differences of the average ΔASAs between training positive samples and negative samples for the former ones (66.9Å^2^) are larger than those for the latter ones (14.5Å^2^). It probably suggests that the larger the difference of the average ΔASAs between training positive samples and negative samples, the better prediction the model yields. In this case, our method also achieves a good prediction. The best MCC among the ten-SVMs is 0.3828 (shown in italics in Table 1). Statistically, the model with threshold 5 makes a good prediction and obtains the highest MCC of 0.4842 (shown in italics in Table 1) after combining the ten-SVMs, comparatively, the 4-th combined SVM achieves the best F1 of 54.62% (shown in bold in Table 1).

**Table 1:** Performance comparison by samples selection by ΔASA

SVM	Sen	Spec	Acc	MCC	Prec	F1	ΔΔ**ASA**
1	24.18	98.31	77.85	0.3728	84.51	37.6	
2	24.11	98.28	77.81	0.3712	84.27	37.5	
3	23.87	98.19	77.67	0.3656	83.41	37.12	66.9Å^2^
4	24.62	98.48	78.09	*0.3828 *	86.04	*38 28*	
5	23.91	98.21	77.7	0.3666	83.57	37.18	

6	20.75	97	75.95	0.2944	72.51	32.26	
7	20.53	96.92	75.83	0.2892	71.75	31.92	
8	20.49	96.9	75.81	0.2883	71.61	31.87	14.5Å^2^
9	21.02	97.1	76.1	0.3003	73.44	32.68	
10**	20.42	96.87	75.77	0.2866	71.35	31.75	

1***	44.58	91.52	78.56	0.4161	66.71	53.45	
2	43.73	93.05	79.44	0.4367	70.59	54	
3	43.03	94.4	80.21	0.4571	74.54	54.56	
4	42.09	95.41	80.69	0.4703	77.77	**54.62**	
5	39.76	96.91	81.14	*0.4842 *	83.07	53.78	
6	6.84	99.17	73.68	0.1726	75.88	12.55	
7	6.16	99.39	73.66	0.1717	79.52	11.44	
8	5.57	99.53	73.59	0.168	81.93	10.41	
9	4.83	99.71	73.52	0.1646	86.37	9.15	
10	4.28	99.82	73.45	0.1611	90.07	8.17	

*The subtraction between average ΔASA for positive samples and that for negative samples.

**The above ten numbers from 1 to 10 stand for the ten-SVMs.

***The following ten ones stand for the combined SVMs with thresholds from 1 to 10.

It is interesting to note that all models perform similarly if the training positive and negative subsets are respectively constructed by random selection without overlap. The details are shown in Table 2 which lists the performance comparison of the ten-SVMs before and after the combination. In this case, the differences of the performance before combining the ten-SVMs are rather small probably due to containing similar average ΔASAs between training positive and negative subsets. Comparison of such performance is meaningless but just listing them here not for ranking. Actually these models also yield good predictions before combining them. In the case of random sample selection, the best MCC among the ten-SVMs is 0.4363 (shown in italics in Table 2). After combining the ten-SVMs, the model with threshold 5 performs better than other models and obtains an MCC of 0.4809 (shown in italics in Table 2), comparatively, the 4-th combined SVM achieves the best F1 of 54.47% (shown in bold in Table 2). Comparison between Table 1 and Table 2 shows that the model with sample selection in the order of ΔASA and that with random sample selection perform similarly after the combination. Moreover, when combining the SVMs, the model performs better and better with threshold from 1 to 5 and, becomes worse and worse with threshold from 6 to 10 as shown in both Table 1 and Table 2.

**Table 2:** Results of predictions by random sample selection

SVM	Sen	Spec	Acc	MCC	Prec	F1
1	34.64	96.77	79.62	0.4338	80.37	48.42
2	34.5	96.72	79.54	0.431	80.03	48.21
3	32.68	97.41	79.54	0.4316	82.78	46.86
4	34.75	96.81	79.68	0.4358	80.62	48.57
5	34.78	96.82	79.7	*0.4363 *	80.68	48.6
6	34.65	96.78	79.63	0.4339	80.39	48.43
7	34.51	96.72	79.55	0.4312	80.06	48.23
8	34.69	96.79	79.65	0.4347	80.48	48.48
9	34.77	96.83	79.7	*0.4363 *	80.69	48.6
10*	34.62	96.77	79.61	0.4334	80.32	48.39

1**	38.49	95.21	79.54	0.4334	75.75	50.93
2	40.61	94.77	79.8	0.4433	74.86	52.6
3	42.47	94.66	80.25	0.4577	75.24	54.26
4	41.79	95.6	80.74	0.4715	78.32	**54.47**
5	40.21	96.62	81.05	*0.4809 *	81.86	53.92
6	29.72	97.7	78.93	0.4107	83.06	43.76
7	28.86	98.12	79	0.414	85.21	43.1
8	28.3	98.33	79	0.4149	86.39	42.62
9	28.13	98.45	79.04	0.4167	87.12	42.52
10	27.72	98.66	79.08	0.4192	88.48	42.2

*The above ten ones are for the ten individual SVMs.

**The following ten ones stand for the combined SVMs with thresholds from 1 to 10.

However, another issue we would like to address is that why the models with random sample selection perform better than those with ΔASAs-sorted sample selection before the combination of classifiers and, surprisingly, why they perform similarly after the combination of classifiers. The reason is probably in that models have been trained efficiently with feasible ΔASAs distribution of training data compared to that of test data. Furthermore, our results suggest that if the ΔASAs distribution of the training data is consistent with that of test data, a good prediction can be yielded.

In addition, the performance comparison under three levels of combined SVMs is listed in Table 3. Among them, the model of combining ten SVMs outperforms that of combining three-SVMs and achieves improvement of MCC by 8.3% and F1 by 8.5%, while the best individual SVM performs the worst among the three cases. It can be concluded that combining outputs of a number of independent classifiers can indeed improve classification rate since the errors made by a classifier can be corrected by the others. However, the best threshold needs to be thoroughly investigated and can be changed in different cases. In this study, the best threshold for 3-combined model is 3 and, 10-combined model with threshold 5 performs the best, when using MCC as a benchmark measurement.

**Table 3:** Prediction results of combined SVMs

SVM	Sen	Spec	Acc	MCC	Prec	F1
Individual*	24.62	98.48	78.09	0.3828	86.04	38.28
3-combined**	31.39	96.74	76.6	0.4009	81.12	45.27
10-combined	39.76	96.91	81.14	0.4842	83.07	53.78

*The best SVM among the ten individual SVMs.

**Average performance when combining three-SVMs selected from the ten-SVMs.

### Prediction performance with the use of SOM

Due to the limitation of residue amount in proteins, adopting more neurons in SOM is not always a good idea to cluster the similar input vectors for residues. Therefore in this work three kinds of SOMs, 3 × 3, 5 × 5, and 7 × 7 SOMs, were investigated. Handling by the two modifications, the relatively less important neurons associated with a small number of samples and those neurons with relatively larger entropies were removed.

**Figure 2:**
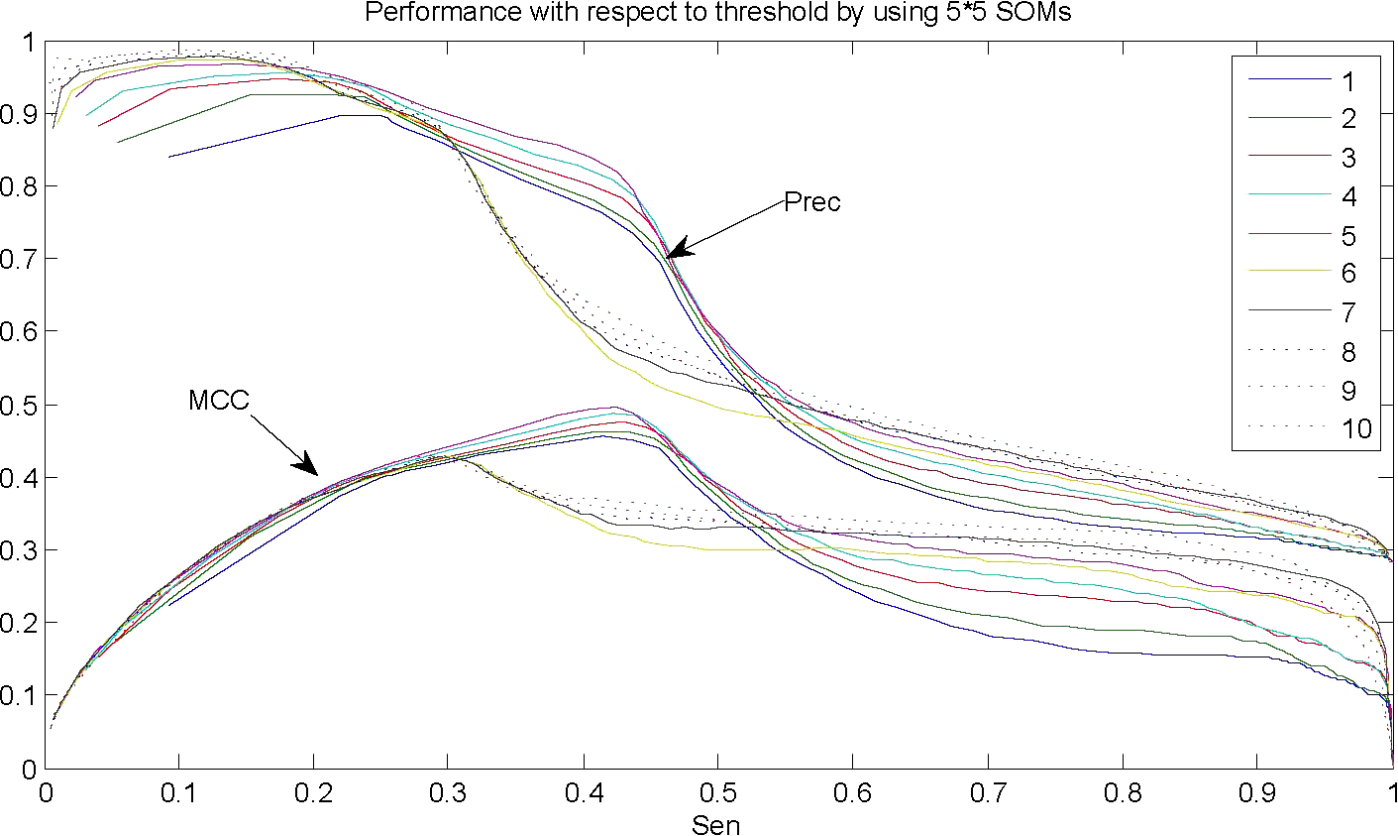
**Performance by our model when using 5 × 5 SOM**. The figure illustrates performance curves of sensitivity-precision and sensitivity-MCC after combining the ten-SVMs. Numbers in the legend stand for SVM with different thresholds. Note that curves with the same color correspond to the model with the same threshold.

In the experiments by using the 5 × 5 SOM and the combination SVM classifiers, we constructed the same 2 × 5 SVM ensembles, trained and tested our model as above. We obtained 25 clusters in total. Clusters from 13 to 17 and clusters from 22 to 25 were retained. Performance by averaging the retained clusters is shown in Figure 2. Results show that the model with threshold 5 outperforms others and achieves the largest MCC of 0.4946 and F1 of 55.95%. Furthermore, it can be found that the 5-th combined SVM performs the best when precision is larger than 50% and, the model with threshold 9 makes the best prediction when sensitivity is larger than 50%. The tendencies of Sensitivity-MCC curves are almost the same as those of Sensitivity-Precision curves.

The model with the 7 × 7 SOM were also constructed and evaluated on the same dataset. A very small improvement was achieved in comparison to the model with the use of 5 × 5 SOM. Table 4 demonstrates the performance comparison among the combined SVMs by the use of the three kinds of SOMs. The model with 7 × 7 SOM outperforms others. It should be noted that the model without SOM also makes a good interface prediction and yields the largest MCC of 0.4842 and F1 of 53.78% as illustrated in Figure 1. Additionally, the case of 3 × 3 SOM by combining three-SVMs is also shown in Table 4, where clusters 1, 2, 3, and 4 with small number of vectors and clusters 5 and 6 with larger entropies were removed. In this case, the model with threshold 3 performs better than that of combining three-SVMs without SOM and makes a small improvement of F1 by 1%, however, it performs much worse (by 8.7% in MCC and 8.3% in F1) than the models with the same SOM by combining the ten-SVMs.

**Table 4:** Evaluation with and without the use of SOM on ensemble of the ten-SVMs

SOM	Sen	Spec	Acc	MCC	Prec	F1
none	39.76	96.91	81.14	0.4842	83.07	53.78
3 × 3*	32.2	96.82	77.2	0.4105	81.46	46.14
3 × 3	40.73	96.68	81.36	0.487	82.17	54.46
5 × 5	42.47	96.35	81.15	0.4946	82.02	55.95
7 × 7	42.84	96.35	81.39	0.4979	81.96	56.25

*Evaluation by combining three-SVMs.

### Improvement by using evolutionary context of residues with respect to hydrophobicity

Kauzmann [44] first pointed out that hydrophobic effect is the most significant property of protein folding and stability. As for the interface prediction, it is often a major contributor to stabilize protein complexes [32]. Gallet et al. proposed a fast method to predict protein interaction sites by analyzing hydrophobicity distribution [18]. This work suggested that interface residues can be identified by using the mean hydrophobicity and the mean hydrophobic moment. However, it appears that the hydrophobic effect alone is insufficient to the protein interface prediction [45] or does not appear to be useful for the interface prediction.

In this work, we used two feature profiles, sequence profile and hydropathy scale. The former was extracted from the HSSP database [46], where each amino acid is represented by elements whose values are based on multiple alignments of protein sequences and their potential structural homologs. The latter was adopted from Kyte-Doolittle's measurement [47]. Despite the two profiles have been used before in interface prediction, the novel integrative technique here can discover the residue's evolutionary context with respect to hydrophobicity in protein-protein interacting sites. It can thus be helpful to improve the interface prediction.

**Figure 3:**
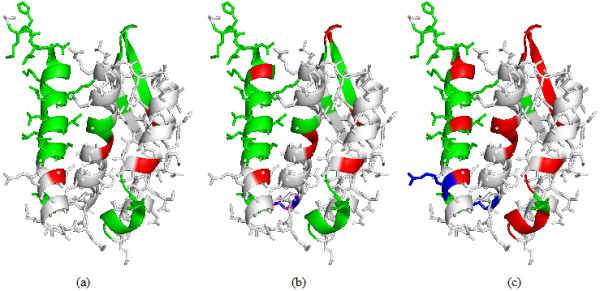
**Comparison between the three profiles on the complex of Bacillus pasteurii urease with acetohydroxamate anion(PDB id: **4UBP**, chain A)**. (a) Prediction results for hydropathy scale; (b) Results for sequence profile; (c) Results for the integrative profile. True prediction interface residues are in red, false predicted non-interface residues are shown in green, false predicted interface residues are in blue, while other ones are in white.

The difference between the integrative profile and each individual profile is that in Equation 4, one profile term would be removed for the model keeping only one profile left. The three pictures in Figure 3 illustrate the interaction identification results by the use of the three profiles: hydrophobic scale, sequence profile, and the integrative profile. From the Figure 3, results show that the model with the integrative profile outperformed the other two, and predicted interface sites more accurately. In addition, the model with sequence profile alone performed better than that with hydropathy scale alone.

To demonstrate the power of the integrative technique, performance for the models with the three profiles are also calculated as discussed above. Table 5 presents the performance comparison for combined SVMs with threshold 5. It can be found that SVM ensembles, whose feature vectors integrate residue sequence profile with hydropathy scale, outperforms the model based on hydropathy scale or sequence profile alone (at least 28% increase in MCC and 8% increase in F1). Moreover, the model with hydropathy scale performs the worst and therefore it cannot be applied to distinguish protein interface residues alone. The performance improvements here indicate that the information contained within the residue sequence profile and the hydropathy scale may be complementary, and that exploiting the complementarity is helpful for predicting protein interface residues.

**Table 5:** Prediction results of ensembles of ten-SVMs with three profiles

Profile	Sen	Spec	Acc	MCC	Prec	F
integrative	42.84	96.35	81.39	0.4979	81.96	56.25
hydropathy scale	9.11	97.61	69.99	0.1505	63.37	15.93
Sequence profile	53.38	68.85	64.02	0.2121	43.74	48.08

### A biological case of improvement by classifier ensemble

Classifier ensemble might perform well in many classifications. Combining the outputs of a number of independent classifiers can improve classification rate since the errors made by a classifier may be corrected by the others [48-50]. Hansen and Salamon [48] denoted that better performance can be achieved by using the optimizational parameters and training different classifiers on different portion of the dataset. In this work, we applied the classifier ensemble technique to combine the outputs from the ten independent SVM classifiers whose training datasets are non-overlapped and thus independent to each other.

**Figure 4:**
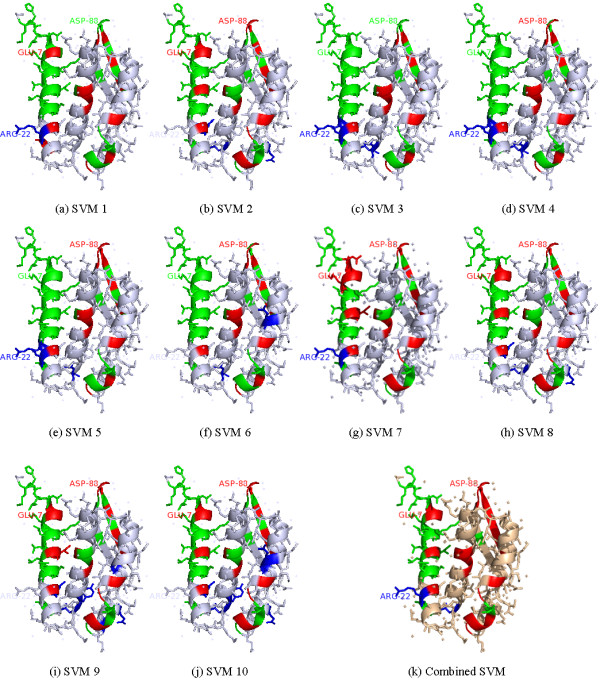
**Performance improvement by the classifier ensemble on the complex of Bacillus pasteurii urease with acetohydroxamate anion(PDB id: **4UBP**, chain A)**. (a)~(j) Prediction results for the ten sub-classifiers; (k) Combined classifier with threshold 5. True prediction interface residues are in red, false predicted non-interface residues are shown in green, false predicted interface residues are in blue, while other ones are in white.

Figure 4 demonstrates the prediction comparison among the ten classifiers and the classifier ensemble. In the case of the model with threshold 5, since interface site ASP-88 (colored in red) is correctly predicted by eight SVM classifiers (except for SVM 1 and SVM 2), the final prediction is correct for this site (see the subgraph (k) in Figure 4). Similarly, five classifiers predict interface site GLU-7 (colored in red) as interface one, thus the site is regarded as interface one finally. Contrastively, six classifiers identify non-interface site ARG-22 (colored in blue) as non-interface site, thus the final prediction for ARG-22 is non-interface site. Therefore, in this case, the prediction errors made by some classifiers can be corrected by the others, resulting in an improvement in performance.

## Discussion

### Comparison with other methods

**Figure 5:**
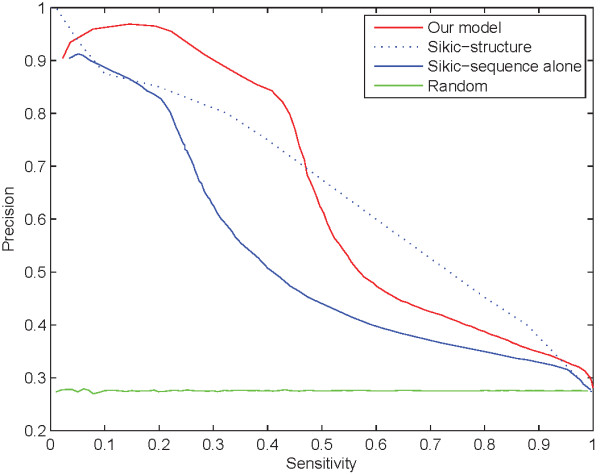
**Comparison with a method in literature **[31]** and a random predictor**. The red line is for our model and the green line is for the prediction of a random predictor, while the blue line and the blue broken line are for the Sikic's method based on sequence alone and based on both sequence and 3D structure, respectively.

Due to different datasets and definitions on interface residues adopted by existing methods, it is very hard to compare prediction performance among different methods. To compare with the current state of the art of protein-protein interaction prediction, we tested on the same dataset and adopted the same definition of interface residues as literature [31], where the dataset was from literature [15]. This dataset consists of 1134 chains in 333 complexes. Figure 5 shows the comparison of sensitivity-precision performance between our model and the Sikic's method based on sequence alone [31]. Additionally, the performance of a random predictor is also affiliated in Figure 5 as reference. In the case of precisions above 90%, our model achieves sensitivities slightly below 30% while Sikic's method achieved sensitivities around only 5%. In the case of precisions from 70% to 80%, Sikic's method achieved a sensitivity level of about 25% while our model reaches sensitivities near to 45%. In these cases our model performs better than Sikic's method based on sequence alone and even better than its prediction based on both sequence and 3D structure (broken line shown in Figure 5). For precisions from 30% to 70%, our model also outperforms Sikic's method based on sequence alone and makes a little worse prediction than that based on both sequence and 3D structure.

Actually, using true secondary structure information and other real 3D structure information in Sikic's method may lead to overestimate the interface predictions, although it obtained a little higher precision than our model with sensitivities from 50% to 90%. Therefore, we just show the performance curve of Sikic's method based on both sequence and real 3D structure, with no purpose of comparison. However, as discussed in Figure 2, our model with threshold 9 performs similar to Sikic's structure-based model when achieving sensitivities from 50% to 90%. It should be noted that our model and Sikic's method share the same definition of interface residues and therefore obtains approximately the same ratio of interface residues to total residues, 27.56% in our dataset and 27.5% in Sikic's method. As a result, our method outperforms Sikic's method based on sequence information. Furthermore, our method based on sequence alone performs similarly to Sikic's method based on both sequence and 3D structure.

Next we discuss the comparison results with other methods whose datasets have different interface fractions, defined as percentages of the total number of protein residues. Table 6 shows the performance comparison of these methods on hetero-complex datasets with sequence alone. In recent years, random forests made a good performance in protein structure prediction, especially in the protein-protein interaction prediction, which is an ensemble method that combines individual classification trees from several bootstrap samples. Chen and Jeong applied random forests in interface prediction and obtained a good F1 of 49% [30], while Sikic *et al*. used random forests and achieved an F1 of 39.7% based on sequence alone and achieved an F1 of 52% based on both sequence and 3D structure information [31]. Our previous work also achieved a good prediction of protein-protein interface residues based on 69 proteins by the use of SVM and evolutionary rates of residues [17]. Note that the comparison aims to demonstrate the development of the protein interaction prediction tools, with no purpose to rank them since predictors were developed based on different datasets, different definitions of interface residues, and different evaluation measurements. Although it is extraordinarily difficult to compare among related methods, our method outperforms others as shown in Table 6. As a result the model by the integrative profile is a very promising approach to predict interface sites.

**Table 6:** Performance of methods on hetero-complexes with sequence alone

Method	Type	Ratio	Sen	Prec	F1
Our model	SVM	27.56	42.84	81.96	56.25
Wang and Chen	SVM	34.8**	61.4	45.8	52.5
Res *et al*.	SVM	16	58.8	26.3	36.3
Koike and Takagi*	SVM	20	28.8	27	27.87
Sikic *et al*.	RF****	27.5	26	84	39.7
Chen and Jeong	RF	10	70	37.7***	49
ISIS *et al*.	NN*****	32*	20	61	30.1
Ofran and Rost	NN	40*	0.5*	62*	0.1

*Based on homo-hetero mixed complexes dataset.

**The ratio of interface residues to surface residues.

***Estimated.

****Random Forests.

*****Neural Network.

### Blind test

To show the potential of our model to practical problem, a CCD-IBD complex (PDB:2bgn) was taken as a test case. Again the evaluation of this blind test is based solely on sequence information without knowing 3D structure of the complex and the true interacting residues.

**Figure 6:**
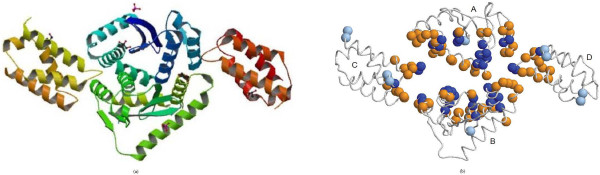
**Visualization of the overall orientation and prediction results on CCD-IBD complex PDB:**2b4j. (a) The overall orientation of CCD-IBD complex; (b) Protein-protein interaction predictions of CCD-IBD complex. The orientation of the complex is illustrated by a smooth spline between consecutive alpha carbon positions. Left graph denotes the natural orientation, while the right one illustrates the protein-protein interaction prediction of the complex. In the right graph, blue sphere stands for TP residue, bluetint one stands for FP residue, and gold sphere demonstrates FN residue. All other residues (not shown as colored spheres) are true negatives (TN). Note that the orientation of the complex in the right graph is varied a little to clearly show the predictions of protein interface residues. Additionally each sphere represents an alpha-carbon atom of each residue. We used RasTop http://www.geneinfinity.org/rastop/ software to display the structure of this complex.

The asymmetric unit of the complex PDB:2bgn contains two molecules, a dimer of integrase (IN) catalytic core domains (CCD) (chains A and B in Figure 6) and a pair of human lens epithelium-derived growth factor (LEDGF) IN-binding domain (IBD) molecules (chains C and D in Figure 6 bound at the CCD dimer interface) [51]. LEDGF binds HIV-1 IN via the small IBD within its C-terminal region. Previous results showed that the IBD is both necessary and sufficient for the interaction with HIV-1 IN [51,52]. There are several key intermolecular contacts at the CCD-IBD interface. Residues Ile365, Asp366, and Phe406 play critical roles in HIV-1 IN recognition as hotspot residues which are located at the interhelical loops within IBD molecules (chain C or D). The water molecule hydrogen-bonds link to the main-chain carbonyl group of LEDGF residue Ile365 and IN residue Thr125. We correctly predict the hotspot residues Ile365 and Asp366. Overall, our method achieves a good prediction performance with a sensitivity of 35.59%, precision of 80.77%, specificity of 96.93%, accuracy of 80.63%, and F1 of 49.41% when achieving the largest MCC of 0.4468. In order for more correct predicted interface residues, our model can obtain a precision of 90.63% with a sensitivity of 27.88%, specificity of 98.84%, accuracy of 78.45%, F1 of 42.65%, and MCC of 0.426. In this case the hotspot residues Ile365 and Asp366 are also predicted correctly.

## Conclusions

This paper addresses the problem of identifying interface residues in hetero-complexes by using an integrative profiling. This novel profile combines residue sequence profile with hydropathy scale and, therefore obtains standard deviation value for each residue in proteins. The deviation value may reveal the evolutionary relationship of a residue in proteins and hydrophobicity in water surroundings. The novel residue profile and an ensemble of SVMs together achieves a good prediction in protein-protein interactions with a sensitivity of 39.76%, precision of 83.07%, specificity of 96.91%, accuracy of 81.14%, and F1 of 53.78% when achieving the largest MCC of 0.4842. In addition, SOM technique is adopted to investigate the interacting relationship of residues. When the SOM technique is used, the prediction performance increases to a sensitivity of 42.84%, precision of 81.96%, specificity of 96.35%, accuracy of 81.39%, and F1 of 56.25% when achieving the largest MCC of 0.4979.

Moreover a residue in our work was represented as a *1-by-19 *vector by using the sliding window with length 19. The scale is much smaller than most other methods. The input vector for representing a residue used in Sikic *et al*.'s method contained *9 *× *20 *= *180 *elements and, 1050 features were used as input vector in Chen and Jeong's method. Therefore our model is very fast and simple. More importantly, a larger number of features in input vectors does not necessarily lead to a better performance. As pointed out by previous work, a machine learning algorithm adopting a simple representation of a sequence space could be much more powerful and useful than using the original data containing all details [53]. Actually biological properties which may be responsible for protein-protein interactions are not fully understood. Therefore how to apply feasible features or feature transformations in protein interaction prediction remains an open problem. Additionally imbalanced data of interface residues and non-interface residues is a very challenging issue, which always causes classifier over-fitting. The ensemble of classifiers may be a feasible pathway to balance training data.

Finally, residue's evolutionary context with respect to hydrophobicity plays an important role in the interface prediction. Above discussion appears to suggest that integrating residue's evolutionary context with other properties of residues, such as residue volume or free energy solution in water, is a plausible way to discover the protein-protein interactions. In our future work, we will investigate the inner relationships of interacting residues, and make use of them for a more accurate prediction.

## Methods

### Data set

The complexes used in this work were extracted from the 3dComplex database [54], which is an database for automatically generating non-redundant sets of complexes. Only those proteins in hetero-complexes with sequence identity ≤ 30% were selected in this work. Meanwhile, proteins and molecules with fewer than 30 residues were excluded from our dataset. Protein chains which are not available in HSSP database [46] were also removed. As a result, our dataset contains 2499 protein chains in 737 complexes. There are mainly two definitions for protein interface residues. The first one is based on differences in ASA of the residues before and after complexation, and the second is based on distance between interacting residues. In this article, the ASA change is used to extract interface residues. We applied the PSAIA software to the extraction [55]. In our case, a residue is considered to be an interface residue if the difference of its ASA in unbound and bound form is > 1Å^2^. As a result, we obtained 142410 interface residues (positive samples) and 374346 non-interface residues (negative samples), where the ratio of the number of positive samples to that of all samples is 27.56%.

In this work we applied a 5-fold cross-validation test to evaluate our proposed method. In this case, proteins in the dataset are divided into 5 subsets which consist of roughly the same number of proteins, one subset is for the test process and the other ones are for the training process.

### Sliding window technique

Similarly to previous works, a sliding window technique is used here in order to involve the association among neighboring residues. It should be noted that the target residue centered on the sliding window plays important role compared to its neighboring ones in the window. Within a sliding window, it is assumed that the influence of residues on the target one fits a normal distribution. Therefore, a series of factors for residues in the window are taken into account to explain how residues affect the probability of the target one being interface residue by using(1)

where *i *is residue separation between residue *x_i _*and the target residue in sequence, *p_i _*denotes an influencing coefficient of residue *x_i _*on the target residue, and *L *is the length of window. *μ *and *σ *are parameters for each residue. In this work, *μ *is regarded as the position of the central target residue and the value is (*L *+ 1)/2, and the standard deviation *σ^2 ^*of residue position is calculated by the following formula:(2)

Then Equation (1) can be rewritten as:(3)

### Generation of residue profiles

It is well known that hydrophobic force is often a major driver to binding affinity. Moreover, interfaces bury a large extent of non-polar surface area and many of them have a hydrophobic core surrounded by a ring of polar residues [56]. The hydrophobic force plays a significant role in protein-protein interactions, however, the hydrophobic effect alone does not represent the whole behavior of amino acids [57]. Therefore, we integrate a hydrophobic scale and sequence profile in the identification of protein-protein interaction residues. In this work, Kyte-Doolittle (KD) hydropathy scale of 20 common types of amino acids is used [47]. Therefore, two vector types are ready for representing residue *i*, one is the KD hydropathy scale vector *KD_i _*and the other one is the corresponding sequence profile *SP_i_*, which is a *1-by-20 *vector evaluated from multiple sequence alignment and the potential structural homologs. Multiplying the two vectors can achieve another 1 × 20 vector for residue *i*. However, representing each residue as a 1 × 20 vector is not always a good idea in residue profiling schema. Here we use a standard deviation of the multiplication to measure the fluctuation of residue *i *in its evolutionary context with respect to hydrophobicity. Then standard deviation value *SD_i _*for residue *i *in a protein is shown as the following form:(4)

where  and  denote the *k*-th value of *SP_i _*and *KD_i _*for residue *i*, respectively, and  denotes the mean value of vector *SP *× *KD*. Note that Equation (4) is an unbiased estimation of . In addition  and  represent the same amino acid type. For instance,  and  all represent residue 'ALA'.

**Figure 7:**
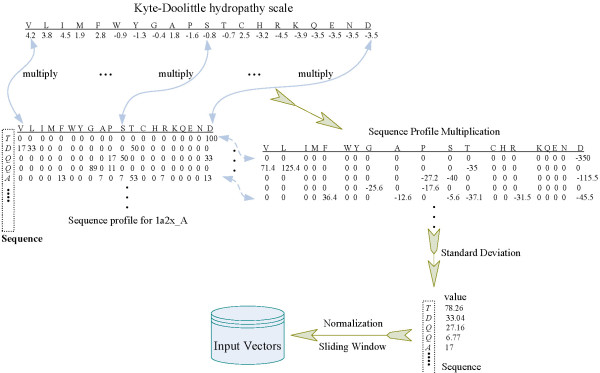
**Flowchart of generating residue profiles**. Each row of the sequence profile corresponds to a residue in the protein, while each column in the sequence profile or the KD hydropathy scale corresponds to each amino acid type.

Furthermore, with a sliding window whose length is an odd number *L*, each residue *i *can be represented as a 1 × *L *vector. The final profile vector for residue *i *in the protein is shown as,(5)

where vector *v_i _*for residue *i *is the multiplication of the standard deviation value *SD_i _*by its influencing coefficient *p_i_*. More details of generating the profile vectors can be referred to an example in Figure 7. For each residue in protein chains, in summary, the input of our model is an array *V_i_*, while the corresponding target *T_i _*is another state value 1 or 0 that denotes whether the residue is located at interface or non-interface region. Similar to most other machine learning methods, our method aims to learn the mapping from the input array *V *onto the corresponding target array *T*. Suppose that *O *is the output from our method, it is trained to make the output *O *as close as possible to the target *T*.

### SVM-SOM classifiers

The number of positive samples or so-called interface residues is much smaller than that of negative samples or non-interface residues. Only 27.56% of the samples are interface residues in this work, which leads to a rather imbalanced data distribution. To overcome this problem, the training positive and negative samples are divided into several subsets without overlap, which have roughly the same sizes, in terms of the order of ΔASA of the corresponding residues before and after complexation. In the case of 5-fold cross-validation test, the positive samples are grouped into two subsets in the order of Δ*ASA *and, the negative samples with Δ*ASAs ≡ *0Å^2 ^are randomly grouped into five subsets due to only a small number of negative samples with 0 < ΔASAs ≤ 1Å^2^.

SVMs are accurate classifiers while they can avoid over-fitting [58,59]. The SVM learner aims to judge whether a residue is located at an interface region or not. As discussed above, there are ten SVMs in the 5-fold test. Here, input profile vector for each residue is extracted as above, and the target value of which is labeled as 1 (positive sample) if the residue is located at interface region and 0 (negative sample) otherwise.

In this study, SOM technique is adopted to group similar input samples and make them more separable [42]. The purpose of SOM is to detect regularities and correlations in their input, and also to recognize groups of similar input vectors. It can adapt their future responses to that input accordingly in such a way that neurons of competitive networks physically near each other in the neuron layer respond to similar input vectors [42]. Readers can be referred to the Additional file 4 for details. Here, we created SOM networks with *N*-by-*N *neurons in a hexagonal layer topology, trained the network on the training set in our dataset by 20 steps, tested proteins on test dataset, and finally obtained *N *× *N *clusters of similar input samples.

Two modifications to the traditional SOM technique are used here, including

• Delete the relatively less important nodes associated with a small number of input samples;

• Use a validation index to choose clusters with the optimal size of the map.

The validation index is adopted from literature [60-62] and presented as an entropy measure. The index is to determine the clusters with the optimal size which can adequately classify the associated input subset without causing overlap. The closer the index value is to 0, the more distinctive the individual categories are. Otherwise the closest index value to the upper bound indicates an absence of any clustering structure in the sample dataset. Therefore we can determine the corresponding clusters with the minimal validation index. Samples in such clusters are then fed into the trained SVMs classifier to identify interface residues. The calculation of the validation index *E *is shown in the following entropy measurement:(6)

Where , *n *= 1, ..., *N*, denotes an input sample, *w_r_*, *r *= 1, ..., *R*, denotes the corresponding weight vector, and *U_rn _*satisfies 0 ≤ *U*_*rn *_≤ 1.

### Classifiers combination

**Figure 8:**
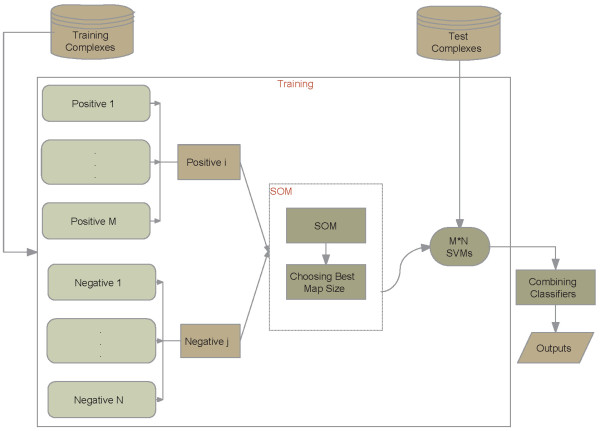
**SVM ensemble for identifying protein-protein interface residues**.

A simple method was used to combine the outputs of SVMs in this paper. A residue was predicted as interface residue if at least *TH *outputs of the SVMs corresponding to the same residue were labeled as positive class 1, otherwise the corresponding residue was identified as non-interface residue. Here *TH*, a threshold value, is ranged from 1 to the total number of SVM classifiers. For example, threshold 2 denotes that one residue was identified as interface residue if at least two outputs of those SVMs were labeled as 1, otherwise as non-interface residue. The flowchart of the whole method is demonstrated in Figure 8. In Figure 8 there are *M *× *N *SVM classifiers, each of which contains balanced training positive and negative input vector sets *i *and *j*.

### Measures for performance evaluation

As discussed in previous literature, there is no single statistic that can adequately assess or rank interface predictors [17,34,63], due to the imbalanced positive and negative datasets. In this work we adopted six evaluation measures to show the performance of our model: sensitivity (Sen), specificity (Spec), accuracy (Acc), precision (Prec), F-measure (F1), and Matthews correlation coefficient (MCC), as defined below(7)

where TP (True Positive) is the number of true positives, i.e., residues predicted to be interface residues that actually are interface residues; FP (False Positive) is the number of false positives, i.e., residues predicted to be interface residues that are in fact not interface residues; TN (True Negative) is the number of true non-interface residues; and FN (False Negative) is the number of false non-interface residues. The MCC is a measure of how well the predicted class labels correlate with the actual class labels. Its value range is from -1 to 1. An MCC of 1 corresponds to the perfect prediction, while -1 indicates the worst possible prediction; an MCC of 0 corresponds to a random guess.

## Authors' contributions

PC carried out the implementation and wrote the manuscript. JYL read and revised the final manuscript. All authors read and approved the final manuscript.

## Supplementary Material

Additional file 1 **Propensity of amino acid types between interface and non-interface sets**. Each histogram is showed in a logarithm (*log*_2_) scale. Click here for file

Additional file 2 **Determination of the sliding window length from the average performance of ensembles of three-SVMs with respect to different window lengths**. The left one shows the average performance with respect to different window lengths for threshold 1 after combining the three-SVMs, while the central and the right graphs are for threshold 2 and threshold 3, respectively. Click here for file

Additional file 3 **Average performance of ensembles of three-SVMs selected from the ten-SVMs**. The left one shows the performance under threshold 1 after combining the three-SVMs, while the central and the right-side sub-graphs are under threshold 2 and threshold 3, respectively. Click here for file

Additional file 4 **Description of SOM**. Click here for file
