# Supplementary material for: Sequence-based identification of interface residues by an integrative profile combining hydrophobic and evolutionary information
Source: BMC Bioinformatics. 2010 Jul 28;11:402. doi: 10.1186/1471-2105-11-402 (PMC2921408; doi:10.1186/1471-2105-11-402)
Supplement: Additional file 1 — Propensity of amino acid types between interface and non-interface sets. Each histogram is showed in a logarithm (log2) scale. [file 1471-2105-11-402-S1.DOC]

Residue composition and propensity

We calculated amino acid composition in our dataset to show the propensity information of the 20 amino acid types between interface and non-interface regions. Each amino acid type belonging to the interface or non-interface set is respectively counted. The composition of the amino acid types involved in the interface or in non-interface set is the fraction of the total number of amino acids involved in interface or in non-interface set to the total amount of each amino acid type. The propensity for each amino acid type is the ratio of the percentage of the type in interface regions to that in non-interface regions. The propensities for the 20 amino acid types in a logarithm (log2) scale are shown in the below figure where the larger the values of residue types, the more possible the residues are located at interface regions, while those with smaller values are more likely located at non-interface regions. Moreover, amino acids with smaller propensity values, such as 'A', 'G', and 'V', representing hydrophobicity, are always involved in non-interface regions. Conversely, hydrophilic amino acids 'R', 'Y', 'W', and 'H' often present in interface regions. Some of these discoveries are consistent with other literature. Interestingly, Arginine is the most frequently occurring residue in interface regions while Cysteine and Alanine appear in non-interface regions mostly.
