# Supplementary figures and images for: Sequence-based identification of interface residues by an integrative profile combining hydrophobic and evolutionary information
Source: BMC Bioinformatics. 2010 Jul 28;11:402. doi: 10.1186/1471-2105-11-402 (PMC2921408; doi:10.1186/1471-2105-11-402)

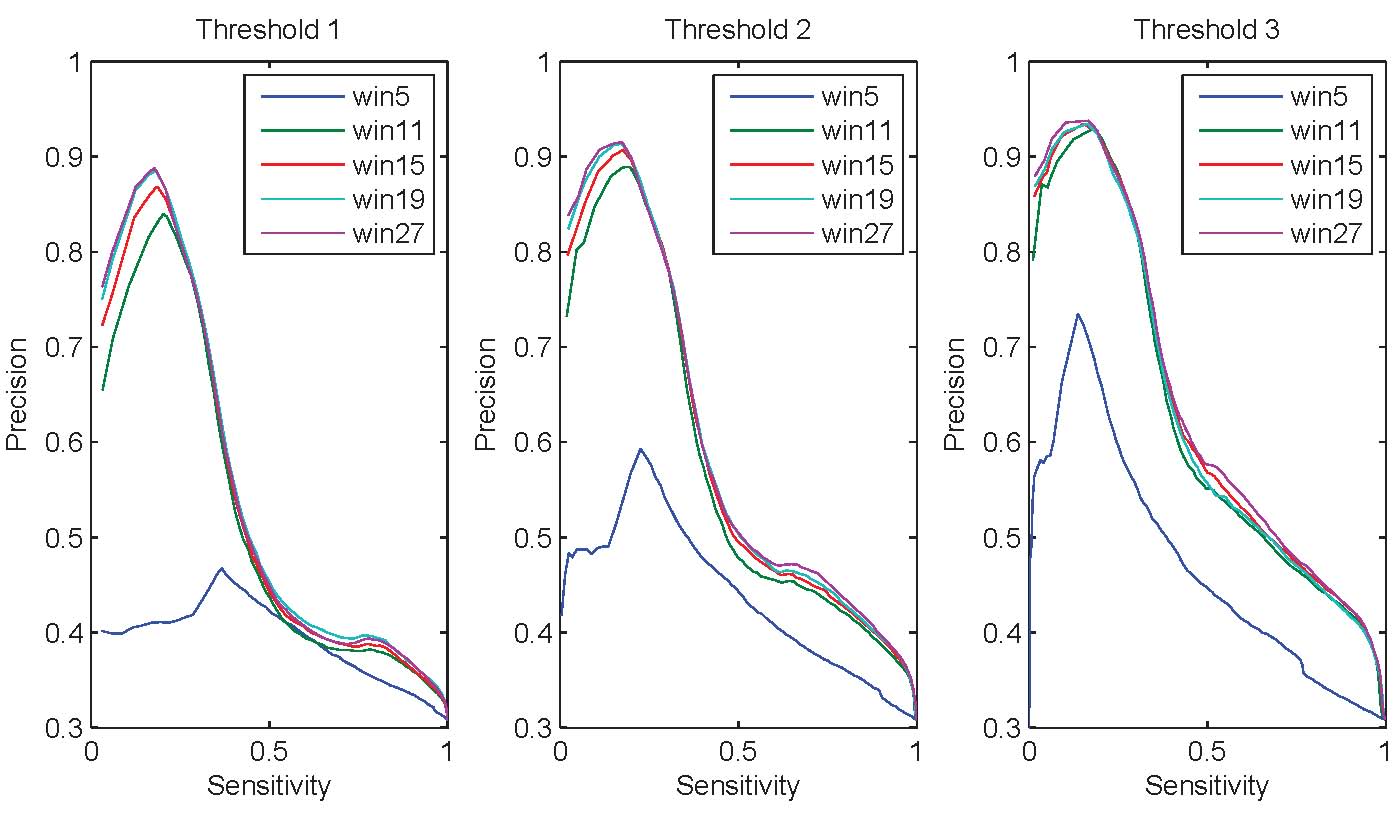

Supplement: Additional file 2 — Determination of the sliding window length from the average performance of ensembles of three-SVMs with respect to different window lengths. The left one shows the average performance with respect to different window lengths for threshold 1 after combining the three-SVMs, while the central and the right graphs are for threshold 2 and threshold 3, respectively. [file 1471-2105-11-402-S2.JPEG]

Threshold 1

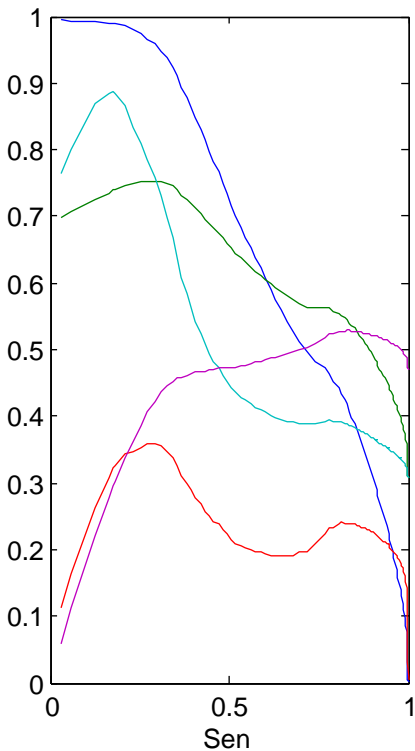

Threshold 2

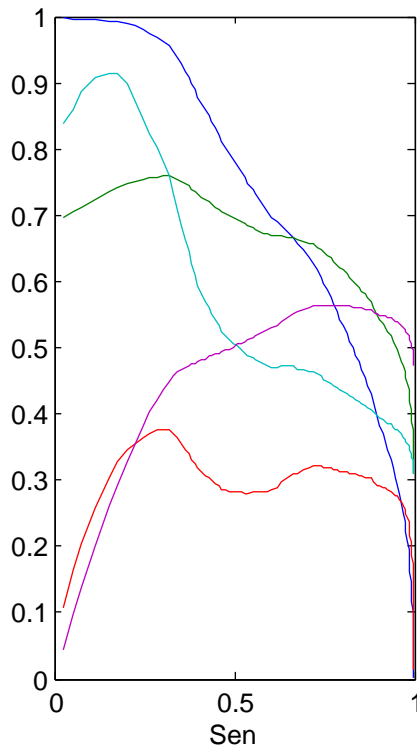

Threshold 3

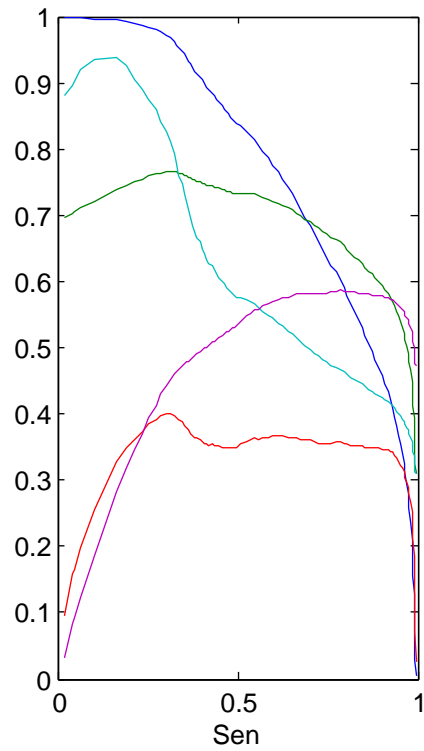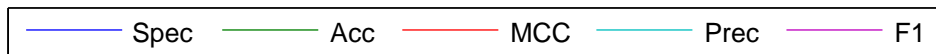

Supplement: Additional file 3 — Average performance of ensembles of three-SVMs selected from the ten-SVMs. The left one shows the performance under threshold 1 after combining the three-SVMs, while the central and the right-side sub-graphs are under threshold 2 and threshold 3, respectively. [file 1471-2105-11-402-S3.PDF]
