# Supplementary material for: Sequence-based identification of interface residues by an integrative profile combining hydrophobic and evolutionary information
Source: BMC Bioinformatics. 2010 Jul 28;11:402. doi: 10.1186/1471-2105-11-402 (PMC2921408; doi:10.1186/1471-2105-11-402)
Supplement: Additional file 4 — Description of SOM. [file 1471-2105-11-402-S4.DOC]

A **self-organizing map (SOM)**, a type of [artificial neural network](http://en.wikipedia.org/wiki/Artificial_neural_network), is trained using [unsupervised learning](http://en.wikipedia.org/wiki/Unsupervised_learning) to produce a low-dimensional (typically two-dimensional), discretized representation of the input space of the training samples, called a map. Unlike to other artificial neural networks, self-organizing map uses a neighborhood function to preserve the [topological](http://en.wikipedia.org/wiki/Topology) properties of the input space.

SOM contains two processes: training and mapping. In training process, it constructs the map using input samples. After the training, it automatically classifiers a new input sample in the mapping process. The map consists of several neurons which associated with a weight vector that has the same dimension as the input sample and a position in the map. The neurons are arranged originally in physical positions according to a topology function, such as a grid, hexagonal, or random topology. The purpose of SOM is to detect regularities and correlations in their input, and also to recognize groups of similar input vectors. It can adapt their future responses to that input accordingly in such a way that neurons of competitive networks physically near each other in the neuron layer respond to similar input vectors.

**Learning algorithm:**

To start the SOM learning, the first step is to initialize all the weights of the neurons either to small random values or sampled evenly from the subspace spanned by the two largest [principal component](http://en.wikipedia.org/wiki/Principal_component) [eigenvectors](http://en.wikipedia.org/wiki/Eigenvectors) of the training samples.

The training utilizes [competitive learning](http://en.wikipedia.org/wiki/Competitive_learning). When a training sample is fed to the network, distances between neurons are calculated from their positions with a distance function. There are several distance functions, such as Euclidean distance, Manhattan distance, and so on. One single winning neuron will be then obtained by calculating the distance between input vector and weight vector. When identifying a winning neuron *i** that is most similar tot the input vector, all neurons within a certain neighbour *Θ (i,q)* of the winning neuron are updated, using the Kohonen rule. The neuron is called the best matching unit (BMU). The weights of the BMU and neurons close to it in the SOM lattice are adjusted towards the input vector at the same time. The magnitude of the change decreases with time and with distance from the BMU. Specifically, all such neuron $i\in\Theta(i*,q)$ are adjusted as follows:

$W_{i}(q+1)=W_{i}(q)+\Theta(i,q)\alpha(q)(p(q)-W_{i}(q))$, (1)

where *α(q)* is a monotonically decreasing learning coefficient and *p(q)* is the input vector.

Thus, when a vector *p* is presented, the weights of the winning neuron and its close neighbors Θ (i,q) move toward input vector *p*. Consequently, after many presentations, neighboring neurons have learned vectors similar to each other. The process is repeated for each input vector for a number of *λ*.

**Algorithm:**

Stepping through the algorithm:

1. Initialize the weight vectors of the map's neurons;
2. Input a sample vector;
3. Traverse each neuron in the map;
4. Use [a distance](http://en.wikipedia.org/wiki/Euclidean_distance) formula to find similarity between the input vector and the weight vector of map's neuron's
5. Track the neuron that produces the smallest distance (this neuron is the best matching unit, BMU)
6. Update the neurons in the neighbourhood of BMU by pulling them closer to the input vector

$W_{i}(q+1)=W_{i}(q)+\Theta(i,q)\alpha(q)(p(q)-W_{i}(q))$,

Where $i\in\Theta(i*,q)$;

1. Increment t and repeat from 2 while *q < λ*.

**Illustration:**

To illustrate the application of SOM technique in bioinformatics, Figure 1 shows the clusters of residues with similar input vectors. The left graph denotes a simple representation of protein chain and the right one denotes the nine clusters using the SOM technique. The figure simply illustrates the mapping of three-dimensional structure of protein into nine clusters. Circles with the same color denote the member of a residue cluster. In the figure, some clusters don’t contain members.


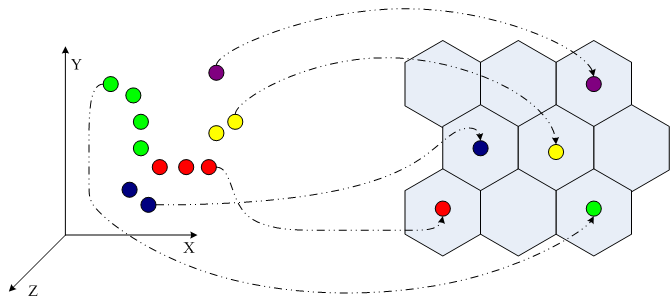


Fig. 1 The mapping of three-dimensional structure of protein into nine clusters using SOM

**Example:**

An example of residues classification for chain ‘A’ in complex 1A2X using SOM is shown in the following figure. Figure 2 plots a SOM layer, where each neuron shows the number of input vectors (corresponding to residues respectively) that it classifies. It shows how many data points are associated with each neuron. The relative number of input vectors for each neuron is shown via the size of a blue colored patch. The SOM was trained with a 3-by-3 set of neurons in a ‘hextop’ topology. The purposes using SOM or other classification techniques are to separate them with designing input vectors as better as possible and to find latent patterns of residues that are significant in protein interactions.

Fig. 2 Illustration of a SOM sample hits for complex chain 1a2x_A. The number centered at each hexagon denotes the amount of residues that associated with corresponding neuron, while the red number denotes the amount of interface residues that are classified into the same cluster.
